# Application and progress of blood flow restriction training in improving muscle mass and strength in the elderly

**DOI:** 10.3389/fphys.2023.1155314

**Published:** 2023-03-24

**Authors:** Junlin Yuan, Li Wu, Ziao Xue, Guodong Xu, Yuxiang Wu

**Affiliations:** Department of Health and Physical Education, Jianghan University, Wuhan, China

**Keywords:** blood flow restriction training, elderly people, muscle mass, muscle strength, muscular atrophy

## Abstract

As an emerging training method, blood flow restriction training has been proved to promote the growth of muscle mass and strength. In recent years, it has been gradually applied in different populations. However, there are few studies on how blood flow restriction training affects muscle mass and strength in the elderly. The relevant literature is compiled and summarized in this study. Through the comparison of blood flow restriction training with traditional training methods and its application in the elderly, it shows that blood flow restriction training can effectively increase muscle mass and strength, prevent muscle atrophy, improve cardiopulmonary function, facilitate injury and postoperative rehabilitation, and intervene in related degenerative diseases as a training method suitable for the elderly,. The main mechanism of blood flow restriction training promoting muscle mass and strength growth is metabolic stress response, including muscle fiber recruitment, protein synthesis signal pathway activation, hormone secretion, etc., and is also related to cell swelling caused by pressure. At present, although the application of blood flow restriction training in the elderly population is increasing, there is a lack of personalized programs. In the future, more research on the dose effect and safety of blood flow restriction training is needed to develop more accurate personalized training programs.

## 1 Introduction

The problem of population aging is becoming more and more prominent, and the proportion of the world’s aging population is increasing. In the World Population Outlook 2019 report, the United Nations noted that the global population is still aging. In 2019, the world’s elderly population over 65 years old will account for one tenth of the total population. In 2050, it will rise to one sixth of the total population, which is the fastest growing period (World Population Prospects, 2019). The arrival of an aging society makes the elderly group more concerned. As people age, muscle mass declines at a rate of 3%–8% every 10 years after the age of 30 (English and Paddon-Jones, 2010). As a result, muscle atrophy and related physical decline are common in the elderly, and the risk of sarcopenia is increased (Centner, et al., 2019). Sarcopenia, the progressive deterioration of muscle mass and strength, is common in the elderly. Prevention of sarcopenia is essential to reduce falls, prevent chronic diseases, and prolong life, and muscle strength training is considered particularly important (Paproski, et al., 2019). At present, it is known that strength training is an effective way to improve muscle mass and strength and prevent muscle atrophy. The American College of Sports Medicine (ACSM) believes that individual repetition of 60%–80% of 1RM resistance can achieve effective gains in muscle hypertrophy and strength. But the older, seriously ill or weak people should start with a lower resistance when they perform resistance training, which may be 40%–50% of 1RM, or even lower (Garber, et al., 2011). However, the elderly and some rehabilitated patients cannot bear the high mechanical pressure exerted on the joints when they perform high-intensity resistance training. And it is easy to cause sports injury in the process of exercise, which is more harmful to the health of the body. Low intensity blood flow restriction training (BFRT), can help the elderly train their musculoskeletal system while keeping the overall intensity low, thereby reducing the risk of sarcopenia (Loenneke and Pujol, 2009).

BFRT also known as pressure training (KAATSU Training), refers to a training method that exerts pressure on human limbs through special pressure devices (such as pneumatic cuff or elastic band) during exercise to block venous return of limbs and reduce arterial blood flow, so as to cause distal ischemia of limbs (Scott, et al., 2015), has been widely used in sports training and rehabilitation therapy as a new training method in recent years. Jeremy P. Loenneke et al. suggest that the combination of BFR and 20%–30% 1RM low-intensity resistance training can significantly increase the volume and strength of muscles and achieve the effect of high-intensity resistance training, while higher intensity load does not bring more benefits (Loenneke, et al., 2012a). Training at an intensity as low as 20% 1RM and combined with blood flow restriction will lead to muscle hypertrophy in just 3 weeks, providing a unique beneficial training mode for promoting muscle hypertrophy (Loenneke, et al., 2010). The characteristic of blood flow restriction training is to use low-intensity exercise load for training, which can also achieve the effect of high-intensity exercise load for training and reduce the risk of sports injury during exercise. Therefore, the blood flow restriction training is more suitable for the elderly people and disease rehabilitation patients, who are prone to sports injury.

The main aims of this review are to investigate the application and progress of BFRT, which improves muscle quality and strength in the elderly. At the same time, it is also compared with two traditional methods of muscle training to discuss the effect, characteristics and advantages of this training method. In addition, the article also discusses the safety and mechanism of blood flow restriction training, which serves as a reference and foundation for the elderly to use safely. Finally, it discusses that blood flow restriction training should be personalized in the future.

## 2 Advantages of blood flow restriction training

High Intensity Resistance Training (HIRT) has been recommended to counteract age-related loss of muscle strength and mass (Chodzko-Zajko, et al., 2009). The American College of Sports Medicine recommends that resistance training of moderate to intense intensity be conducted at least twice a week to maintain or enhance muscle strength and quality (Kraemer, et al., 2002). However, some elderly people are often unable to perform high-intensity exercise, and the heavy load involved in HIRT is unlikely to be suitable for the entire elderly group, including the elderly without training experience, the elderly with a weak body, and the elderly with dysfunction.

Some researchers believe that for the same muscle or muscle group, healthy adults can effectively improve muscle quality and strength by performing BFR combined with low-intensity resistance training 2–3 times a week (Pope, et al., 2013). Felipe C Vechin et al. compared the effects of low-intensity resistance training combined with BFR and high-intensity resistance training on the quality and strength of quadriceps femoris in the elderly. The test results of body composition changes showed that the two training schemes could effectively improve the cross-sectional area (CSA) of quadriceps femoris and the 1RM of leg push. Both low-intensity blood flow restriction training and high-intensity resistance training had the effect of increasing muscle strength and skeletal muscle quality, and there was no significant difference between the two groups (Vechin, et al., 2015). The mechanism of muscle hypertrophy can be caused by mechanical stress and metabolic stress. Traditional high-intensity resistance training mainly causes muscle hypertrophy through mechanical stress, while low-intensity pressure resistance training can cause muscle hypertrophy through metabolic stress (Pearson et al., 2015). A case report pointed out that BFR combined with low-intensity resistance strength training improved the strength and skeletal muscle mass of 91 years old osteoporosis patients. However, these effects were not detected after alone strength training of the same intensity (Lopes, et al., 2019). These results suggest that BFRT should be considered as an alternative clinical intervention to prevent muscle loss and improve the functional health of the elderly osteoporosis population, because it is often difficult to control the exercise program of HIT in these populations.

The results of an experiment on the effect of high-intensity intermittent walking training on the physique of the middle-aged and elderly people conducted by the Medicine School of Shinshu University show that high-intensity intermittent walking training is beneficial to strengthening the leg muscle strength of the elderly, and some high-intensity components can be encouraged to be added to the sports guidance of the healthy elderly (Nemoto, et al., 2007). Victor Silveira Coswig et al. took elderly women as subjects and conducted high-intensity intermittent training twice a week for 8 weeks. The results showed that the body mass, fat content, body fat percentage and resting blood pressure decreased significantly. After the training, these decreases still remain compared with the baseline (Coswig, et al., 2020). Another study reported that after 10 weeks of high-intensity intermittent walking training for the elderly with rheumatoid arthritis, the changes in body composition were not obvious. The same thing was that the resting heart rate and blood pressure were significantly reduced, and the cardiopulmonary function was improved (Bartlett, et al., 2018). It shows that the elderly may only choose walking training when performing high-intensity interval training (HIIT), because it is difficult for them to perform high-intensity resistance training, so when the elderly chooses HIIT, the main goal may be to improve aerobic capacity and cardiopulmonary function, rather than muscle strength.

In conclusion, HIRT, HIIT and BFRT all have the effect of enhancing muscle strength. However, as shown in Table 1, compared with HIIT, HIRT and BFRT have a more obvious effect on improving muscle strength and mass. However, compared with HIRT, BFRT has a unique advantage in that it achieves the effect of increasing muscle mass with a very light load. For the elderly, this is a good method because they may not be able to complete the muscle training with heavy load such as HIT. Therefore, BFRT can be used as a reasonable and effective method to train the muscle strength of the elderly.

**TABLE 1:** Comparison of the effects of different training methods on muscle.

Training method		HIIT^①^	HIRT^②^	BFRT^③^
Training type		intermittent walking	resistance training	resistance training
Training intensity		high	≥70%1RM	20%–30%1RM
Training effect	**muscle mass**	Increased but not significantly	increased	increased
	**muscle strength**	Increased but not significantly	increased	increased
	**aerobic capacity**	increased	—	—

HIIT^①^: High-intensity Interval Training, HIRT^②^: high intensity resistance training, BFRT^③^: blood flow restriction training.

## 3 Application of blood flow restriction training in the elderly

Due to the particularity of the elderly, the application of blood flow restriction training in this field mainly includes strength training to prevent muscle atrophy and muscle function decline, and it also includes sports rehabilitation for many chronic diseases and rehabilitation treatment after sports injury or joint surgery.

### 3.1 Application of blood flow restriction training in muscle strength training of the elderly

Strength training is usually based on resistance training. Some researchers have proved through experiments that healthy elderly men through 20% 1RM resistance training combined with BFR and traditional high-intensity 80% 1RM resistance training, after 6 weeks of planned training, the leg muscle strength increased significantly, and there was a very similar strength adaptation between the two groups (Karabulut, et al., 2010). BFRT not only directly increases the muscle mass and strength of the elderly, but also adapts to it after the withdrawal of training. Tomohiro Yasuda reported that the increased muscle strength of the elderly after 12 weeks of training with BFRT was well maintained after 24 weeks of detraining (Yasuda, et al., 2014a). Therefore, BFR combined with low-intensity resistance training is applied to the strength training of the elderly, providing a practical and effective method for the elderly, and the effect of this training method has a certain continuity.

In addition, Matthew John Clarkson et al. have found that BFR combined with simple low-intensity walking training can improve the muscle strength and physical function of the sedentary elderly and the clinical population characterized by decreased physical function (Clarkson, et al., 2017). 10 weeks of BFR combined with walking training can increase the thigh muscles and improve the aerobic capacity of the elderly (Ozaki, et al., 2011b). BFR combined with walking training improved the chair standing performance of older women, which tended to be related to knee strength. The improvements of functional fitness may be mainly due to increases in strength as measured by significant increases in maximal isometric and isokinetic knee joint torques (Ozaki, et al., 2011a). Besides, BFRT is also widely used in strength recovery training and postoperative rehabilitation training. Some researchers took RA patients as experimental subjects. After 12 weeks of planned training and supervision, it is proved that BFRT can effectively improve the muscle strength, quality and function of RA patients, and the pain feeling caused by exercise and the score of health assessment questionnaire are better than those of non-pressure group. BFRT can become a feasible treatment method in RA management (Rodrigues, et al., 2020). BFR combined with intensity resistance training can safely improve the lower limb strength and function of Parkinson’s disease (PD) patients, and reduce the discomfort of Restless Legs Syndrome (RLS), which is conducive to the rehabilitation of patients and improve the quality of life (Douris, et al., 2022). A trial conducted by the United Kingdom National Health Service showed that patients with anterior cruciate limb reconstruction (ACLR) underwent BFRT leg push training after operation, and the knee joint pain during and after exercise was lower than that of traditional resistance training. BFRT may be more beneficial in the early stage of ACLR postoperative rehabilitation (Hughes, et al., 2019). Shuoqi Li et al. have studied the effect of blood flow restriction training on muscle strength and pain in knee joint injuries, and the results show that BFR combined with low-load resistance training (LLRT) and high-load resistance training (HLRT) can significantly improve muscle strength, but the pain score after L-BFR is significantly decreased (Li, et al., 2021). Similar studies evaluated whether BFR combined with LLRT could significantly reduce Anterior Knee Pain (AKP) compared with LLRT alone. The blood flow restriction group alleviated the pain of functional activities immediately after the intervention, and the effect was significant, while the group without blood flow restriction had no effect (Korakakis, et al., 2018). Therefore, L-BFR is a potential intervention that can be applied to the rehabilitation of knee joint injury. Clinically, it means that BFR may be a more reliable method to reduce pain. Another case report observed that strength training with a low BFR of 65 mmHg was able to induce a positive health effect on a 90-year-old man, with a great improvement in muscle quality and strength (Lopes, et al., 2019).

In conclusion, BFR combined with low-intensity resistance training can effectively increase the muscle mass and strength of the elderly. Additionally, low-intensity walking training combined with BFR for the elderly tends to improve the strength of thigh muscles and knee joints, which can effectively prevent muscle atrophy, improve activity capacity, and lower the risk of falling due to insufficient muscle strength, even though the effect of muscle growth is not as good as that of resistance training. In addition, BFRT can be used as an effective intervention in strength training for postoperative rehabilitation.

### 3.2 Application of blood flow restriction training in the treatment of chronic diseases in the elderly

With the increase of age, the locomotor apparatus in the body will age and degenerate, which are manifested as osteoporosis, muscle relaxation, joint stiffness, slow movement of the whole body, etc., and even some people have a series of physical diseases, such as diabetes and hypertension, due to improper living habits or overwork when they are young (Vandervoort, 2002). BFR treatment, as a treatment method, has been paid more and more attention and used in sports medicine (Baker, et al., 2020). Therefore, as a new training method, blood flow restriction training is increasingly applied to the rehabilitation treatment of some elderly chronic diseases.

One study reported that BFRT was performed every 2 weeks, and after 8 weeks, insulin levels decreased and insulin sensitivity increased (Kambič, et al., 2019). I. Satoh et al. found that after 3 months of BFRT treatment in patients with metabolic syndrome, Glycated Hemoglobin (HbA1c) decreased by 10% and low-density lipoprotein cholesterol decreased by 8% (Satoh, 2011). Although these results do not directly demonstrate the effect of BFRT on patients with Type 2 Diabetes (T2D), BFRT may improve metabolic control in patients with T2D by improving muscle mass and muscle metabolism and reducing insulin levels (Saatmann, et al., 2021). Antonio Crisafulli et al. have shown that after 1 month of blood flow restriction training, the blood pressure of healthy adults at rest before and after training has not changed, but the blood pressure decreases during training, which indicates that BFRT may have a hypotensive effect (Crisafulli, et al., 2018). Relative ischemia caused by blood flow restriction during exercise can reduce the pressor reflex during exercise (Sundblad, et al., 2018). Experiments have observed that compared with BFR, heart rate and diastolic blood pressure in the non-BFR program increased significantly after exercise, and the cardiovascular response values at the end of all exercise in the BFR group did not change significantly (Kacin and Strazar, 2011). The changes of these hemodynamic indexes are within the normal range, so this method is considered to be safe and feasible in some elderly and disease patients (Neto, et al., 2017).

To sum up, the existing research proves that BFR combined with low-intensity resistance training can be applied to a variety of chronic diseases that are prone to occur in the elderly, and provides effective rehabilitation treatment for some elderly people and patients with diseases. And because the exercise intensity of BFRT is low, it will not produce high mechanical pressure on the joints, which is a safer method for the elderly.

## 4 Safety and feasibility analysis of blood flow restriction training

In recent years, as the use rate and attention of blood flow restriction training in the fitness field and competitive sports field have gradually increased, its safety and feasibility have also attracted much attention. Compared with traditional training, there are more variables in blood flow restriction training, such as blood flow restriction degree (cuff width and restriction pressure) and training variables (training intensity, amount, frequency and interval time) may affect the effect of blood flow restriction training, improper use may also cause safety problems (Nakajima, et al., 2011). Therefore, the safety and feasibility of blood flow restriction training has always been a concern.

### 4.1 Safety of blood flow restriction training to restriction pressure

Restriction pressure is one of the important factors that affect the safety of blood flow restriction training. Different restriction pressures may produce different responses. Marcelo Conrado de Freitas et al. proposed that when blood flow restriction training with lower load intensity (10%–20% 1RM) is performed, the effect of increased restriction pressure on muscle growth is more obvious (de Freitas, et al., 2017). However, excessive or inappropriate pressure application during blood flow restriction training may lead to venous thrombosis, and long-term improper use may also lead to venous injury and hardening (Nakajima, et al., 2011). Therefore, in order to ensure the safety of training, appropriate limiting pressure should be used during blood flow restriction training. The pressure value of KAATSU training was first put forward by Yoshiko Sato, the inventor of KAATSU training, and has been continuously improved to become the commonly used pressure standard (see Table 2).These standards are formulated according to the changes of blood flow of different people under quiet and exercise conditions. Relevant studies mainly measure the specific resting systolic pressure (SBP) and resting arterial occlusive pressure (AOP) of different subjects to conduct personalized pressure setting as a reference, and the pressure range is usually between 40% and 80% AOP(Counts, et al., 2016; Kim, et al., 2017). In addition, it should be noted that these pressures are 3cm and 5 cm respectively for the width of the upper and lower limb compression bands. When a wider compression band is used, the compression pressure should be appropriately reduced.

**TABLE 2:** Comparison table of binding pressure and inflation pressure for different groups of people.

	Target	Binding pressure (mmHg)	Upper limit charging pressure (mmHg)	Pressurization time (min)
**Upper limb**	elderly people	20–30	60	3
	middle-aged and elderly	20–30	90	6
	ordinary people	30–40	120	9
	fitness crowd	30–40	150	12
	physical athlete	40–50	180	15
	elite physical athlete	40–50	220	18
**Lower limbs**	elderly people	20–30	90	6
	middle-aged and elderly	30–40	120	9
	ordinary people	30–40	150	12
	fitness crowd	40–50	180	15
	physical athlete	40–50	210	18
	elite physical athlete	50–60	250	21

### 4.2 Safety of cardiovascular system

Because blood flow restriction training affects blood flow, people often worry that venous congestion and dilation caused by blood flow restriction may lead to venous valve damage during blood flow restriction training (Loenneke, et al., 2011a). Especially for special groups such as the elderly and the rehabilitation population, the safety of the cardiovascular system is the focus of research.

Previous studies have reported potential negative effects of BFRT, such as increased incidence of blood clots, venous congestion/dilation, ischemia-reperfusion injury, muscle injury, and strain rhabdomyolysis (Loenneke, et al., 2011b; Hughes, et al., 2017; Patterson, et al., 2019). However, it was concluded that compared with traditional training, blood flow restriction training is unlikely to bring additional risks in most cases (Cristina-Oliveira, et al., 2020). In addition, some safety studies on BFRT pointed out that no evidence of increased thrombotic risk was found in the post BFR rheology study (Heitkamp, 2015). Another study observed experimentally that the arterial blood flow of the subjects was not completely blocked during the blood flow restriction training, and the stroke output slightly decreased, but not the cardiac output. After the pressure was relieved, the stroke output immediately returned to the normal level, which may be part of the reason for the safety of the blood flow restriction training widely used in muscle training (Iida, et al., 2007). Another study showed that when aerobic exercise combined with BFR training in the elderly, compared with the control group without BFR, the increase in mean blood pressure, systolic blood pressure, and diastolic blood pressure was greater, but there was no significant difference, and the resting-state blood pressure after exercise can be reduced, which is an achievable and beneficial choice for the elderly (Staunton, et al., 2015). These existing studies suggest that some of the variables present during blood flow restriction training may be factors that increase or decrease the potential risk.

To sum up, the study on the influence of blood flow restriction training on cardiovascular system shows that various parameters are controlled within a scientific and reasonable range during the training process, and blood flow restriction training is safe and feasible in most cases. Although these existing evidences do not show the adverse effects of blood flow restriction training on the cardiovascular system, for the elderly population or patients with cardiovascular diseases with vulnerable cardiovascular system, a more safe and accurate pressure range is needed to determine its safety.

## 5 Mechanism of blood flow restriction training

Although researchers have confirmed the effect of blood flow restriction training on promoting muscle quality and strength, its mechanism of action is not clear. Different scholars have explained the possible mechanism of blood flow restriction training from different angles. Most of the current research results point to the interaction of various potential mechanisms of action, mainly including muscle fiber recruitment, protein synthesis signal pathway activation. Metabolic stress reactions such as hormone secretion, and cell swelling caused by blood flow restriction, which make the muscles have adaptive responses.

### 5.1 Metabolic stress

During blood flow restriction training, metabolites accumulated in large amounts due to restricted blood flow in the limbs (Yasuda, et al., 2014b), stimulation of afferent nerve fibers and inhibition of α Motor neurons, thereby increasing the recruitment of muscle fibers to maintain muscle strength (Loenneke, et al., 2011a, Hwang et al., 2019). According to the size principle of muscle fibers, slow muscle fibers are the first to be used by muscles during exercise. With the continuous increase of exercise intensity, the recruitment of fast muscle fibers with high threshold is constantly increased (Henneman, et al., 1965). Due to the rapid accumulation of lactic acid and other metabolite products, nerve fatigue is caused more quickly, and the human nervous system mistakenly thinks that it has entered a very intense exercise. Therefore, more muscle fibers are stimulated, so that the muscle fibers are rapidly recruited (Kubota, et al., 2011). It has achieved the effect of high-intensity training, and early investigation by Jeremy P. Loenneke et al. have proved that the increase of fast muscle fiber recruitment is an important factor to promote muscle growth (Loenneke, et al., 2011b). However, a study on weightlifters showed that after low-intensity BFRT, muscle hypertrophy mainly occurred in type Ⅰ muscle fibers (Bjornsen, et al., 2019). This may be due to the interference caused by the ability of the testers before the test, and scholars have different views on this mechanism.

Muscle growth ultimately stimulates protein synthesis by increasing synthesis or reducing metabolic signaling pathways (Pearson et al., 2015). The Mammalian Target of Rapamycin (mTOR) is considered to be a key factor in regulating skeletal muscle growth. It is involved in the regulation of messenger Ribonucleic Acid (mRNA) translation initiation and plays an important role in exercise-induced muscle protein synthesis and training induced hypertrophy (O'Neil, et al., 2009). During muscle training, insulin-like growth factors-1 (IGF-1) activates protein kinase B (aka Akt) to stimulate protein translation by inducing mTOR, which is involved in the regulation of mRNA translation initiation, and plays an important role in exercise-induced muscle protein synthesis and training induced hypertrophy (O'Neil, et al., 2009; Bodine, et al., 2001). Other studies showed that after low-intensity resistance exercise combined with blood flow restriction training, skeletal muscle protein synthesis was stimulated, and the phosphorylation levels of ribosomal S6 kinase (S6K1) and ribosomal protein S6 (rpS6) were significantly increased, which enhanced the signal of MTOR translation and thus increased muscle synthesis (Fujita, et al., 2007; Fry, et al., 2010; Gundermann, et al., 2014). In addition, due to the increase of intracellular Ca2+ concentration during exercise, neuronal Nitric Oxide synthase-1(NOS-1) is activated and nitric oxide (NO) is produced, which can directly activate the mTOR signaling pathway to promote protein synthesis (Ito, et al., 2013). The production of NO may activate muscle satellite cells, which proliferate and differentiate and fuse with each other to form muscle fibers, resulting in hypertrophy of muscle fibers (Anderson, 2000; Snijders, et al., 2015). In addition, Shouyu Xu et al. have found that BFRT can not only enhance the signal pathway to promote protein synthesis, but also inhibit muscle decomposition by reducing the expression of ubiquitin proteasome and myostatin, thus achieving the effect of preventing muscle atrophy and promoting muscle hypertrophy (Xu, et al., 2016).

Previous studies have shown that the increased concentration of anabolic hormones such as Growth-Hormone (GH) and IGF-1 after strength training is one of the important mechanisms that trigger muscle hypertrophy and plays a crucial role in muscle growth (Kraemer and Ratamess., 2005; Fink, et al., 2018). Greg V Reeves et al. compared low intensity resistance exercise with blood flow restriction and moderate intensity resistance exercise without blood flow restriction. Later, they believed that the increase of hormone secretion after blood flow restriction training could mediate muscle hypertrophy. (Reeves, et al., 2006). In the process of blood flow restriction training, due to the accumulation of a large number of metabolites, the pH of the internal environment decreases, and the low pH environment induced by metabolic accumulation stimulates GH secretion (Loenneke, et al., 2011a; Yasuda, et al., 2014a; Hwang and Willoughby 2019). It has been reported that blood flow restriction training makes the GH level after exercise 10 times higher than that of the control group without blood flow restriction (Reeves, et al., 2006). Another study found that the GH concentration increased after 20% 1RM BFR knee extension training, up to 290 times that in the resting state (Loenneke, et al., 2012c). Many scholars believe that GH mainly induces the whole process of skeletal muscle hypertrophy by enhancing the release of IGF-1 (Schoenfeld, 2013). Studies have shown that the increased concentration of GH stimulates the release of IGF-1 from the liver (Loenneke, et al., 2012b). IGF-1 is considered to be a growth factor, and the increase in IGF-1 protein levels has been shown to be proportional to the increase in resistance training muscle strength (Kostek, et al., 2005). The study also found that the concentration of IGF-1 was significantly increased after blood flow restriction training, which proved that this mechanism may play a role in blood flow restriction training (Abe, et al., 2005; Madarame, et al., 2010).

### 5.2 Cell swelling

It has been reported that a novel mechanism among the mechanisms by which BFRT triggers muscle hypertrophy is the increase of intracellular hydration, a phenomenon known as cell swelling (Pearson and Hussain et al., 2015). Some scholars have suggested that cell swelling can induce changes in protein anabolism and catabolism, and cell swelling can inhibit catabolism and shift protein balance to anabolism (Haussinger, et al., 1993). Other studies have shown that besides inhibiting catabolism, cell swelling can also positively affect metabolism by saving protein and promoting lipolysis (Berneis, et al., 1999; Keller, et al., 2003). Although the potential anabolic mechanism of cell swelling is still unclear, it is conceivable that during the blood flow restriction training, the blood accumulation and the accumulation of metabolites induced by the blood flow restriction may be sufficient to cause changes in the water balance inside and outside the cell and increase the pressure gradient inside and outside the cell (Loenneke, et al., 2012a). Some studies have found the phenomenon of cell swelling after blood flow restriction training (Yasuda, et al., 2012). However, little research has been done on this mechanism so far, and it has not been demonstrated whether the inhibition of catabolism due to cell swelling is beneficial for muscle growth after blood flow-limiting training.

**FIGURE 1:**
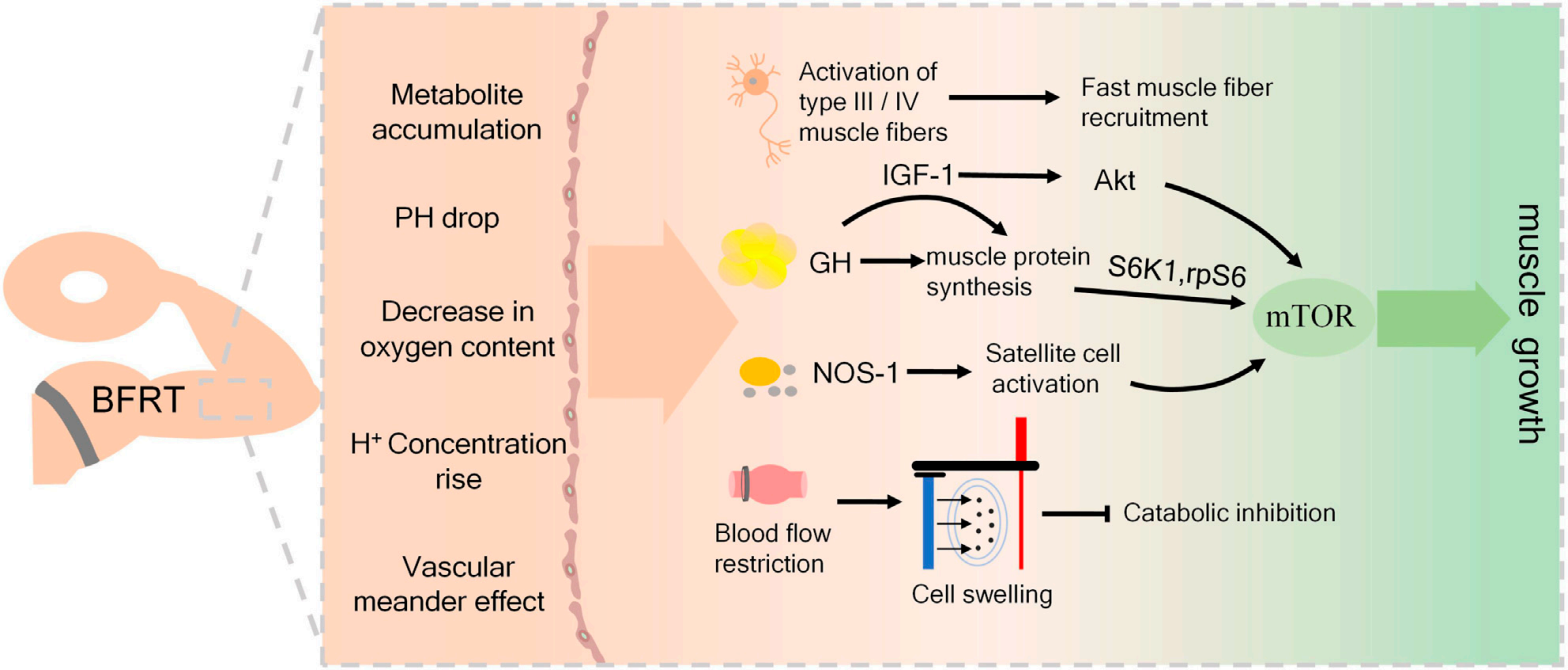
Mechanism of blood flow restriction training.

To sum up, although the use of BFRT is indeed effective for muscle strength growth and muscle hypertrophy, the mechanism supporting muscle adaptation by BFRT is not very clear. Limited evidence indicates that metabolic stress activates these unclear mechanisms to a large extent, mainly including muscle fiber recruitment, hormone secretion, protein signaling pathway activation and cell swelling, and ultimately promotes muscle protein synthesis, as shown in Figure 1. However, a more in-depth investigation is necessary to determine the specific mechanisms responsible for the positive effects of BFRT, and to ascertain the impact of related factors on the activation of muscles.

## 6 Discussion

Muscle strength is the basis for people to participate in various physical activities. With the increase of age, the quality and strength of human muscles decline or even atrophy which is an important reason for falls and injuries. Strength training is a method to effectively improve muscle mass and strength while preventing muscle atrophy. It can also prevent injuries and enhance sports ability. Traditional strength training usually requires an intensity of more than 70% 1RM to achieve effective benefits in muscle strength and muscle hypertrophy. However, not every variety of people can use and achieve such high training intensity. Combining BFRT with low-intensity resistance training can significantly improve muscle strength and muscle mass in older adults. Similarly, combining BFRT with aerobic exercise can significantly improve muscle endurance and cardiorespiratory function, ultimately leading to improved quality of life in older adults (Staunton, et al., 2015). Although the safety of BFRT has been questioned, based on the current extensive investigations in short-term use, there is still no evidence that BFRT leads to the formation of blood clots or causes damage to the cardiovascular system and muscles (Clark, et al., 2011; Iida, et al., 2011; Madarame, et al., 2013; Pinto, et al., 2018; Bond, et al., 2019). However, it is important to note that most studies have been conducted on healthy adult populations, and there is limited research on individuals with cardiovascular diseases. Therefore, in the long-term use of this training method, it is recommended that older adults and individuals with cardiovascular diseases seek guidance from healthcare professionals and undergo medical supervision to ensure safe and effective implementation. Further research is needed to determine the long-term effects of BFRT on individuals with different health conditions. Currently, BFRT is increasingly being applied to the elderly population. However, it is important to note that the application of BFRT to the elderly lack’s personalization, as different individuals may have different adaptability to pressure and resistance. In the future, it is necessary to develop more reasonable and refined training schemes that are based on the characteristics and needs of different individuals. This approach will optimize the benefits of BFRT for older adults and help them achieve their individual fitness goals.

## 7 Conclusion

BFRT can achieve the effect of high-intensity resistance training with low and medium intensity resistance training. It can be used by a wide range of people, especially for the elderly and rehabilitation groups. Existing studies show that BFRT can effectively increase muscle mass and strength. Combining BFR with low-intensity resistance training can be an effective strategy to prevent disuse muscle weakness, muscle atrophy, and sarcopenia resulting from aging and prolonged periods of inactivity. Additionally, it can facilitate patient rehabilitation and promote the recovery and treatment process. BFRT is a promising and versatile training method that has the potential to benefit a wide range of individuals. Further research is needed to optimize its application and to fully understand its potential benefits and mechanism.
